# Epidemiology of birth defects based on surveillance data from 2011–2015 in Guangxi, China: comparison across five major ethnic groups

**DOI:** 10.1186/s12889-018-5947-y

**Published:** 2018-08-13

**Authors:** Jichang Chen, Xuemei Huang, Bo Wang, Yu Zhang, Chokechai Rongkavilit, Dingyuan Zeng, Yongjiang Jiang, Ba Wei, Chawla Sanjay, Eric McGrath

**Affiliations:** 1 Maternal and Child Health Care Hospital, 50 Yingshan Street, City Central District, Liuzhou, Guangxi 545001 People’s Republic of China; 2 Department of Family Medicine and Public Health Sciences, Division of Behavioural Sciences, 6135 Woodward Ave. I-Bio Building Room1127, Detroit, MI 48202 USA; 3 Valley Children’s Hospital, Madera, California, 93720 USA; 4 Division of infectious Diseases, Children’s Hospital of Michigan, Wayne State University School of Medicine, 3901 Beaubien Blvd, Detroit, MI 48201-2119 USA

## Abstract

**Background:**

The causes of birth defects (BDs) are complex and include genetic and environmental factors and/or their interactions. More research is needed to describe the epidemiology of BDs within specific regions of China. This study focused on differences in the prevalence of BDs based on ethnicity in a large city in Guangxi Province, China.

**Methods:**

Surveillance data of infants born in 114 registered hospitals in Liuzhou between 2011 and 2015 were analyzed to determine the epidemiology of BDs across five major ethnic groups. We calculated the prevalence of BDs and relative risk of BDs by ethnicity.

**Results:**

There were 260,722 perinatal infants of which 6581 had BDs, with the average prevalence of 25.24 per 1000 perinatal infants (PIs). Prevalence data showed an obvious uptrend over the past 5 years. Han had the highest prevalence of total BDs (28.98‰), followed by Zhuang (25.19‰), Yao (18.50‰), Miao (15.78‰) and Dong (14.24‰). Relative to the Han; Zhuang, Miao, Yao, and Dong had a lower risk of musculoskeletal and urogenital malformations; Miao and Yao had a lower risk of cardiovascular malformation; and Dong had a lower risk of cardiovascular and craniofacial malformation. Several maternal risk factors were found to be associated with BDs (e.g., maternal and gestational age, number of antenatal care visits).

**Conclusion:**

This study provided a comprehensive description of ethnic differences in the risk of BDs in Liuzhou City, China. Observed ethnic differences in the risk of BDs may be related to genetic susceptibilities, environment, cultural customs, or to potential combinations of these factors.

## Background

The World Health Organization estimates that approximately 260,000 deaths (7% of all neonatal deaths) globally were caused by birth defects (BD) s in 2004 [1]. It is estimated that the prevalence rates of BDs is 4.7% in the developed countries, 5.6% in the middle-income countries, and 6.4% in the low-income countries [1, 2]. China is a middle-income country with the largest population in the world. With 16 million births annually, it is expected that there are 0.9 million BDs each year in China [3]. Based on the most recent surveillance data in 2011, BDs have become the second most common cause of infant deaths in China (the leading cause being premature/low birth weight) [3–5]. BDs are the main causes of spontaneous abortion, stillbirth, perinatal death, infant death and congenital disability. BDs also affect the child’s and the family’s quality of life and carry a significant economic burden to the family and the society, particularly in the setting of China’s one-child and recently two-child policy since 2013 [3, 6, 7].

The causes of BDs are complex and multifactorial. Nearly 50% of BDs cannot be ascribed to a specific cause [8, 9]. In 1964, after the thalidomide tragedy, several countries including the Great Britain, Israel, and Finland started conducting BD surveillance [10–14]. The Chinese Ministry of Health started the BD surveillance systems in 1986 [15–17]. The surveillance was initially paper-based data reporting method, that was replaced by an electronic, web-based reporting system developed by the National Office for Maternal and Child Health Surveillance in 1998 [18]. The quality of birth defect monitoring varies in different parts of the world, and may vary even within the same country or region [19, 20]. In China, the emphasis placed on monitoring for BDs is higher and more comprehensive in Eastern China than in Midwestern China [3].

Guangxi is one of the western provinces of China, and the Chinese minorities account for majority of the population in Guangxi province (37.2%) [21]. Liuzhou is the most representative city in Guangxi, for its geographic (population of 3.8 million), economic (region’s industrial center), composition of population (large ethnic minority groups), and culture and customs within the ethnicities [22]. There are fifty-six (56) ethnic groups in China, and more than thirty ethnic groups reside in Liuzhou. The largest ethnic group in Liuzhou is Han (48.9%), followed by Zhuang (35.2%), Miao (6.4%), Dong (6.3%), Yao (1.9%), and Mulao (0.8%) [21, 23, 24]. The Chinese ethnic groups have their own, different languages, culture, customs and living environments. The epidemiology of BDs among various Chinese ethnic groups has not been described in the literature. Since the etiology of BDs could be multi-factorial involving genetic factors, environmental factors and their interactions, it is crucial to understand the difference in BD phenotypes among Chinese ethnic groups. Therefore, our study aims to provide further insights on BDs among the different Chinese ethic groups in Liuzhou. The information gained may allow clinicians to provide proper counselling to families, allow public health officials to appropriately plan targeted interventions, and may provide the foundation for further etiologic and epidemiologic studies.

## Methods

### Disease classification

The diagnosis of BD was based on the “International Statistical Classification of Diseases and Related Health Problems, Tenth Edition” (ICD-10) and Chinese National Criteria of BDs [25]. BDs were categorized by the system affected, including neurologic system, craniofacial system, gastrointestinal system, urogenital system, musculoskeletal system, cardiovascular system, respiratory system, genetic metabolic diseases, genetic syndrome, and other BDs (i.e., those BDs do not belong to the above-mentioned 9 systems). BDs were classified into ten systems (including neurologic, craniofacial, gastrointestinal, urogenital, musculoskeletal, cardiovascular, respiratory, genetic metabolic, genetic syndrome, and other) in our study. When a patient had BDs affecting ≥2 organ-systems, then he/she was categorized as having multiple BDs.

### Data collection

All surveillance data of BDs were collected from the obstetrics departments or neonatal departments according to the “National Office for Maternal and Child Health Surveillance” formulated by the National Health and Family Planning Commission. The surveying population was perinatal infants (including stillbirth, fetal death or live birth between 20 weeks of gestation through 7 days after birth) born in the 114 registered hospitals of Liuzhou from 2011 to 2015. In total, infants were followed six times in the first year of life. The schedule for follow-up monitoring was as follows: Within 7 days for the first time-point, and then at 6 weeks, 3 months, 6 months, 8 months and 12 months old. Infant BDs were reported to the monitoring system when diagnosed within 7 days of life.

Each delivery that was associated with a BD was reported using a registration card for BDs submitted by physicians in obstetrics and gynecology, pediatric or neonatal medicine through an online hospital-based survey, required by the Chinese government. Each case report card recorded basic maternal information, (including ethnicity, residence, family income, education, mother’s age, number of antenatal care visits, gestational age at first antenatal care visit, number of reported abortions, pregnancy outcomes, Human Immunodeficiency Virus (HIV), Hepatitis B Virus (HBV), and syphilis infections), birth information (e.g., gestational age, weight of birth), diagnoses of specific BDs, symptoms, medication use during early pregnancy, and family history. In addition to the case report card, a quarterly table for each registered hospital was completed by professional physicians. Each quarterly table contained 3 months of data such as the number of perinatal births, maternal age, residence, ethnicity, occupation, pregnancy history, gestational age of birth, gestational age at first antenatal care attendance, number of antenatal visits, infant gender, number of BDs, and maternal illness.

Both case report cards and quarterly tables were reviewed and audited by maternal and child health hospitals and health administrative departments. Periodic quality control measures were in place at the monitored hospitals and occurred quarterly at the county-level and bi-annually at the city-level or province-level to assure reporting accuracy.

### Approval for this study was obtained from institutional review Board of Liuzhou Maternal and Child Health Hospital

#### Statistical analysis

The prevalence rates and 95% confidence intervals of overall BDs were calculated for the whole sample and stratified by the five major ethnic groups across all years (2011–2015) of the study. A line chart was then constructed to graphically display the longitudinal trends of the prevalence of BDs by the different ethnic groups. The prevalence rates of the top fifteen BDs were also calculated and compared among the six major ethnic groups using chi-square tests. Multiple logistic regression analysis was performed to further examine the association of ethnicity with BDs, controlling for potential confounders including maternal age, gestational age, number of antenatal care visits, gestational age at first antenatal care visit, number of previous abortion, and syphilis infection. The dependent variable of logistic regression analysis was whether the infant had a birth defect (no/yes). Only variables identified as significantly associated with the dependent variable at *P* < 0.05 in the bivariate analyses were included in the model. Adjusted odds ratios (ORs) and their 95% confidence intervals (CIs) were calculated. All statistical analyses were performed using the SAS 9.4 statistical software package (SAS Institute Inc., Cary, NC, USA). A significance level of 0.01 was adopted in bivariate comparisons and multivariate analysis due to the large sample size.

## Results

### Study sample

A total of 260,722 perinatal infants were monitored from 2011 to 2015. Of the perinatal infants, the top 5 ethnic groups were represented as 44.7% were Han, 36.6% were Zhuang, 7.5% were Miao, 6.9% were Dong, and 2.4% were Yao (see Table 1). The rest, 1.8%, included other various, small ethnic groups which are not further discussed.

**Table 1:** Ethnic distribution in Liuzhou’ population (Percent) and prevalence rate (per 10,000 Perinatal Infants) of total birth defects

	Han	Zhuang	Miao	Dong	Yao
Percent of population	44.73	36.64	7.53	6.92	2.41
Prevalence rate of birth defects	28.98	25.19	15.78	14.24	18.45

### Prevalence and trend of total BDs

Of the 260,722 perinatal infants, 6581 had BDs, with an average prevalence of 25.2 (95% CI: 24.6–25.9) per 1000 PIs (perinatal infants) (Table 2). Trend analysis revealed that the annual prevalence rates of total BDs in the 5 years increased linearly (χ^2^ trend = 59.92, P < 0.0001). The total prevalence rate increased by 1.49% annually from 2011 to 2015 on average.

**Table 2:** Prevalence rates of total birth defects in Liuzhou from 2011 to 2015

Year	Perinatal infants	birth defects	Prevalence of birth defects per 1000 Perinatal Infants (95% CI)	Fixed base growth rate (%)
2011	36,344	863	23.75(22.20–25.37)	–
2012	58,744	1084	18.45(17.38–19.58)	22.31
2013	54,474	1571	28.84(27.44–30.29)	21.43
2014	55,925	1595	28.52(27.15–29.95)	20.08
2015	55,235	1468	26.58(25.24–27.96)	11.92
total	260,722	6581	25.24(24.64–25.86)	–

*CI* confidence interval

### Ethnic comparisons of total BDs

Han had the highest prevalence rate of total BDs in our study (29.0 per 1000), followed by Zhuang (25.2 per 1000), Yao (18.5 per 1000), Miao (15.8 per 1000) and Dong (14.2 per 1000) (Table 3). Compared with Han (as the reference group), the risk of total BDs was lower in Zhuang (RR = 0.87, CI 0.83–0.92), Miao (RR = 0.54, CI 0.49–0.61), Dong (RR = 0.49, CI 0.43–0.56), and Yao (RR = 0.64, CI 0.53–0.76) (Table 4).

**Table 3:** Ethnic distribution of each year Liuzhou’ birth defects from 2011 to 2015

Year	Han	Zhuang	Miao	Dong	Yao
2011	24.25	25.49	24.27	16.02	13.83
2012	25.92	13.57	13.09	7.87	10.38
2013	33.22	29.13	14.77	16.48	27.54
2014	31.71	29.61	18.76	16.91	20.15
2015	29.27	28.94	12.30	15.41	18.92

**Table 4:** Ethnic distribution of total Birth defects in Liuzhou from 2011 to 2015

Birth defects	Han		Zhuang		Miao		Dong		Yao
	No.	No.	RR (CI)	No.	RR (CI)	No.	RR (CI)	No.	RR (CI)
Total birth defects	3380	2406	0.87(0.83–0.92)^c^	310	0.54(0.49–0.61)^c^	257	0.49(0.43–0.56)^c^	116	0.64(0.53–0.76)^c^
Multiple birth defects	216	192	1.09(0.89–1.32)	22	0.60(0.39–0.94)^a^	20	0.60(0.38-0.95)^a^	11	0.94(0.52-1.73)
Genetic synd/meta	105	84	0.98(0.73–1.30)	6	0.34(0.15–0.77)^a^	9	0.55(0.28-1.09)	1	0.18(0.02–1.27)
Birth defects prevalence^§^	28.98	25.19		15.78		14.24		18.45	
Perinatal infants	116,626	95,524		19,645		18,049		6288	

*CI* confidence interval, *RR* relative risk, *Multiple* birth defects affecting ≥2 organ-systems, *synd/meta* syndrome and metabolic disease

^§^= Prevalence per 1000 perinatal infants

^a^*P* < 0.05, b *P* < 0.01, c *P* < 0.001

From 2011 to 2015, 89 classes (or combinations of classes) of BDs in total were found in our sample, and the top fifteen classes of BDs were musculoskeletal malformation, cardiovascular, craniofacial, urogenital system, other BDs, neurologic, gastrointestinal system, genetic syndrome, respiratory system, musculoskeletal with cardiovascular systems, cardiovascular with other BDs, cardiovascular with urogenital systems, cardiovascular with craniofacial systems, musculoskeletal with other BDs, and craniofacial with musculoskeletal systems. Relative to the Han, Zhuang had significantly lower risks in musculoskeletal and urogenital system malformation (RR = 0.83, CI 0.75–0.93; RR = 0.66, CI 0.57–0.77, respectively); Miao and Yao had significantly lower risks in musculoskeletal system (RR = 0.74, CI 0.60–0.92; RR = 0.62, CI 0.42–0.92), cardiovascular system (RR = 0.30, CI 0.21–0.41; RR = 0.58, CI 0.39–0.87), urogenital malformation (RR = 0.27, CI 0.18–0.42; RR = 0.47, CI 0.26–0.83) and other BDs (RR = 0.60, CI 0.43–0.82; RR = 0.54, CI 0.31–0.97); Dong had statistical significant lower risks in the musculoskeletal system (RR = 0.66, CI 0.52–0.83), cardiovascular system (RR = 0.22, CI 0.15–0.32), craniofacial system (RR = 0.73, CI 0.54–1.00), urogenital malformation (RR = 0.24, CI 0.15–0.39) and other BDs (RR = 0.55, CI 0.35–0.72) (see Table 4). The top six birth defect classes comprised over 85% of the total BDs (see Table 5).

**Table 5:** Top fifteen classes of total Liuzhou’ Birth Defects from 2011 to 2015, and ethnic distribution

Birth defects	Han		Zhuang		Miao		Dong		Yao
	No.	No.	RR (CI)	No.	RR (CI)	No.	RR (CI)	No.	RR (CI)
Musculoskeletal	774	528	0.83(0.75–0.93)^c^	97	0.74(0.60–0.92)^b^	79	0.66(0.52–0.83)^c^	26	0.62(0.42–0.92)^a^
Cardiovascular	741	604	1.00(0.89–1.11)	37	0.30(0.21–0.41)^c^	25	0.22(0.15–0.32)^c^	24	0.58(0.39–0.87)^b^
Craniofacial	405	279	0.84(0.72–0.98)	46	0.67(0.50–0.91)	46	0.73(0.54–1.00)^a^	16	0.73(0.44–1.21)
Urogenital	477	259	0.66(0.57–0.77)^c^	22	0.27(0.18–0.42)^c^	18	0.24(0.15–0.39)^c^	12	0.47(0.26–0.83)^b^
Other group birth defects	409	278	0.83(0.71–0.97)	41	0.60(0.43–0.82)^b^	32	0.50(0.35–0.72)^c^	12	0.54(0.31–0.97)^a^
Neurologic	153	115	0.92(0.72–1.17)	25	0.97(0.64–1.48)	17	0.72(0.44–1.18)	12	1.45(0.81–2.62)
Gastrointestinal	85	67	0.96(0.70–1.33)	12	0.84(0.46–1.53)	10	0.76(0.39–1.46)	2	0.44(0.11–1.71)
Genetic syndrome	92	60	0.80(0.58–1.10)	4	0.26(0.09–0.70)	7	0.49(0.23–1.10)	1	0.20(0.03–1.45)
Respiratory	26	17	0.80(0.43–1.47)	3	0.69(0.21–2.26)	3	0.75(0.23–2.46)	0	0.35(0.02–5.74)
Mus & Cardio	21	25	1.45(0.81–2.60)	1	0.28(0.04–2.10)	0	0.15(0.01–2.48)	1	0.88(0.12–6.57)
Cardio & other	22	20	1.11(0.61–2.03)	0	0.13(0.01–2.17)	1	0.29(0.04–2.18)	1	0.84(0.11–6.25)
Uro & Cardio	19	14	0.90(0.45–1.79)	1	0.31(0.04–2.33)	0	0.17(0.01–2.74)	1	0.98(0.13–7.29)
Cran & Cardio	20	9	0.55(0.25–1.21)	0	0.14(0.01–2.39)	4	1.29(0.44–3.78)	0	0.45(0.03–7.48)
Mus & Other	9	13	1.76(0.75–4.13)	3	1.98(0.54–7.31)	2	1.44(0.31–6.65)	2	4.12(0.89–19.07)
Cran & Mus	8	13	1.98(0.82–4.79)	3	2.23(0.59–8.39)	0	0.38(0.02–6.59)	0	1.09(0.06–18.90)
Rest Birth Defects	119	107	1.10(0.85–1.43)	15	0.75(0.44–1.28)	13	0.71(0.40–2.12)	6	0.94(0.41–2.12)
Perinatal infants	116,626	95,524		19,645		18,049		6288	

*CI* confidence interval, *RR* relative risk, *Mus* Musculoskeletal, *Cardio* Cardiovascular, *Cran* Craniofacial, *Uro* Urogenital, *gensyn* genetic syndrome, *resp* respiratory

a P < 0.05, b P < 0.01, c P < 0.001

The prevalence rates of top five BDs within musculoskeletal malformation, cardiovascular, craniofacial, urogenital, neurologic and other malformation were compared across five large ethnic groups (Table 6). With regards to musculoskeletal malformation, Zhuang, Miao, and Dong had lower prevalence of talipes equinovarus and Yao had a lower prevalence of pigmented nevus than Han. Three cardiovascular-specific risks (patent foramen ovale, patent ductus arteriosus and atrial septal defect) were less prevalent in Miao and Dong compared to Han. Congenital ear anomaly and genital anomaly were less prevalent in Zhuang, Miao, Dong, and/or Yao. Whereas hydrocephalus was more prevalent in Yao compared to Han. In addition, angioneoplasm was less prevalent in Zhuang and Miao and brain anomaly was less prevalent in Dong.

**Table 6:** Highest five prevalences of specific birth defects from the top six system classifications of total Liuzhou' birth defects from 2011 to 2015, and ethnic distribution

Birth defects	Han		Zhuang		Miao		Dong		
	No.	No.	RR (CI)	No.	RR (CI)	No.	RR (CI)	No.	RR (CI)
Polydactylism or Symphysodactylia	457	338	0.90(0.78–1.04)	70	0.91(0.71–1.17)	52	0.74(0.55–0.98)^a^	16	0.65(0.39-1.07)
Talipes equinovarus	175	103	0.72(0.56–0.92)^b^	17	0.58(0.35–0.95)^b^	15	0.55(0.33–0.94)^a^	5	0.53(0.22-1.29)
Limb reduction	40	30	0.92(0.57–1.47)	4	0.59(0.21–1.66)	6	0.97(0.41–2.29)	1	0.46(0.06–3.37)
Pigmented nevus	16	8	0.61(0.26–1.43)	1	0.37(0.05–2.80)	1	0.40(0.05–3.05)	3	0.47(0.26–0.83)^b^
Pectus or rickets	15	14	1.14(0.55–2.36)	0	0.19(0.01–3.20)	0	0.21(0.01–3.48)	0	0.60(0.04–10.00)
Patent foramen ovale	127	95	0.91(0.70–1.19)	5	0.23(0.10–0.57)^b^	7	0.36(0.17–0.76)^b^	5	0.73(0.30–1.78)
Patent ductus arteriosus	104	108	1.27(0.97–1.66)	4	0.23(0.08–0.62)^b^	0	0.03(0.00–0.50)^a^	2	0.36(0.09-1.44)
Atrial septal defect	90	66	0.90(0.65–1.23)	2	0.13(0.03–0.54)^b^	6	0.43(0.19–0.98)^a^	1	0.21(0.03-1.48)
Ventricular septal defect	72	59	1.00(0.71–1.41)	5	0.41(0.17–1.02)	4	0.36(0.13–0.98)^a^	2	0.52(0.13-2.10)
Atrial septal defect & Patent ductus arteriosus	59	55	1.14(0.79–1.64)	2	0.20(0.05–0.82)^a^	3	0.31(0.10-1.00)^a^	1	0.31(0.04-2.27)
Ear anomaly	212	133	0.77(0.62–0.95)^a^	19	0.53(0.33-0.85)^b^	12	0.37(0.20–0.65)^c^	6	0.52(0.23–1.18)
Cleft lip with or without palate	167	130	0.95(0.76–1.20)	24	0.95(0.76–1.20)	32	1.24(0.85–1.81)	9	1.00(0.51–1.95)
Eye anomaly	14	9	0.78(0.34–1.81)	0	0.20(0.12–3.43)	1	0.46(0.06–3.51)	0	0.64(0.04–10.72)
Ear anomaly & Cleft lip with or without palate	3	0	0.17(0.01–3.38)	2	3.96(0.66–23.69)	0	0.92(0.05–17.87)	1	6.18(0.64–59.43)
Other craniofacial	2	2	1.22(0.17–8.67)	1	2.97(0.27–32.74)	0	1.29(0.06–26.92)	0	3.71(0.18–77.25)
Genital anomaly	221	106	0.59(0.46–0.74)^c^	7	0.19(0.09–0.40)^c^	6	0.18(0.08–0.39)^c^	4	0.33(0.12–0.90)^a^
Kidney anomaly	107	56	0.64(0.46–0.88)^b^	10	0.55(0.29–1.06)	6	0.36(0.16–0.82)^a^	1	0.17(0.02-1.24)
Hypospadias	102	65	0.30(0.19–0.46)^c^	2	0.12(0.03–0.47)^b^	5	0.32(0.13–0.78)^a^	4	0.73(0.27-1.97)
Polycystic kidney disease	25	12	0.59(0.29–1.17)	1	0.24(0.03–1.75)	1	0.26(0.04–1.91)	2	1.48(0.35–6.26)
Oystic kidney disease	6	5	1.02(0.31–3.33)	1	0.99(0.12–8.22)	0	0.50(0.03–8.82)	1	3.10(0.37–25.67)
Schridde syndrome/thalassaemia	201	135	0.82(0.66–1.02)	26	0.77(0.51–1.16)	22	0.71(0.46–1.10)	6	0.55(0.25–1.25)
Angioneoplasm	66	32	0.59(0.39–0.90)^c^	0	0.04(0.00–0.72)^a^	4	0.39(0.14-1.07)	4	1.12(0.41–3.08)
Lymphadenoma	43	39	1.11(0.72–1.71)	2	0.28(0.07–1.14)	2	0.30(0.07–1.24)	0	0.21(0.01–3.46)
Omphalocele or Gastroschisis	43	30	0.85(0.53–1.36)	8	1.10(0.52–2.35)	1	0.15(0.02–1.09)	1	0.43(0.06–3.13)
Other cavity effusion	22	10	0.56(0.26–1.17)	3	0.81(0.24–2.70)	1	0.29(0.04–2.18)	1	0.84(0.11–6.25)
Brain anomaly	55	42	0.93(0.64–1.93)	5	0.54(0.22–1.35)	2	0.24(0.06–0.96)^a^	3	1.01(0.32-3.23)
Hydrocephalus	36	22	0.75(0.44–1.27)	9	1.48(0.72–3.08)	7	1.26(0.56–2.82)	6	3.09(1.30–7.33)^a^
Anencephaly	18	14	0.95(0.47–1.91)	3	0.99(0.29–3.36)	4	1.44(0.49–4.24)	2	2.06(0.48–8.88)
Spinal bifida or Neural tube defect	14	7	0.61(0.25–1.52)	2	0.85(0.19–3.73)	1	0.46(0.06–3.51)	0	0.64(0.04–10.72)
Encephalocele	5	14	3.42(1.23–9.49)^a^	0	0.54(0.03-9.76)	0	0.59(0.03–10.62)	0	1.69(0.09–30.49)
Perinatal infants	116,626	95,524		19,645		18,049		6288	

*CI* confidence interval, *RR* relative risk

^a^*P* < 0.05, ^b^ *P* < 0.01, ^c^ *P* < 0.001

### Associations of prevalence of BDs with perinatal characteristics

Results of the multivariate logistic regression analysis indicate that ethnicity was significantly associated with BDs. Compared to Han, ethnic minorities (Zhuang, Miao, Dong and Yao) had lower risk of overall BDs. Mother’s age (> 35 years), gestational age (< 36 weeks), a higher number of antenatal care visits (12 or more), and a higher number of reported abortions (8 or more) were significantly positively associated with overall BDs (Table 7).

**Table 7:** Odd ratios from multiple logistic regression analysis showing predictive factors for birth defects among 260,722 Chinese perinatal infants

Characteristics	aOR	95%CI	*P* value
Ethnic group			
Zhuang	0.86	0.81~0.91	< 0.0001
Miao	0.56	0.50~0.63	< 0.0001
Dong	0.52	0.46~0.60	< 0.0001
Yao	0.64	0.53~0.78	< 0.0001
Han (ref)	1.00		
Mother’s age			
< 20 years	0.82	0.70~0.97	0.0371
20~24 years	0.81	0.73~0.90	< 0.0001
25~29 year	0.87	0.79~0.96	0.0037
30~34 years	0.91	0.82~1.00	0.0577
35 years or above	1.00		
RPR test for syphilis infection			
Positive	1.42	1.05~1.93	0.0229
Negative (ref)	1.00		
Gestational age (weeks)			
Less than 34	9.82	8.93~10.80	< 0.0001
34~36	2.50	2.27~2.75	< 0.0001
37 weeks or more (ref)	1.00		
Number of antenatal care visits			
< 4 times	1.02	0.93~1.11	0.7182
5~8 times	0.82	0.76~0.90	< 0.0001
9~11 times	0.76	0.70~0.83	< 0.0001
12 times or more (ref)	1.00		
Gestational age at first antenatal care visit			
≤ 9 weeks	1.00	0.90~1.10	0.9789
10~14 weeks	0.98	0.88~1.10	0.7984
≥ 15 weeks			
Number of previous miscarriages/spontaneous abortions^38,39^			
< 3 times	0.48	0.22~1.05	0.0647
4~7 times	0.35	0.15~0.78	0.0100
8 times or more (ref)	1.00		
Gender of perinatal infants			
Male	1.27	1.20~1.35	< 0.0001
Female (ref)	1.00		

*ref* reference, *CI* confidence interval, *aOR* adjusted Odd Ratio

## Discussion

Our study performed a comprehensive analysis of ethnic differences in the perinatal infant prevalence of BDs in Liuzhou, Guangxi, China over a 5 year time period. The average prevalence rate of total perinatal infants’ BDs was 25.2 per 1000 PIs in the past 5 years, with an upward trend increasing by 1.49% on average each year. The ethnic distribution in our study sample was consistent with the ethnic distribution in Liuzhou, Guangxi as reported in the 2012 Chinese population census [21, 23].

The average prevalence rate (25.2 per 1000 PIs) was higher than 20.2/1000, which was reported in a prior analysis of BDs in perinatal children in Guangxi from 2001 to 2010 [26]. The upward trend in the prevalence rate of BDs has been noted commonly in recent epidemiologic BD studies in China [3, 18, 25–28]. The upward trend in BD epidemiology is potentially explained by several factors. Firstly, there is continued development of laws and regulations in Maternal and Child Health Care that have led to increased focus on BD surveillance and there is now full development of a government-led online BD surveillance system for the entire country. There is continued improvement in the overall health awareness of the country along with significant national financial support used in projects targeting tertiary prevention against BDs (pre-marital medical examination, pre-pregnancy care, genetic counselling, family planning, education on optimal reproductive age, and folic acid supplementation), along with the wider use of prenatal diagnosis techniques and the timely and effective diagnosis (e.g., Thalassemia carrier screening in pre-marital and pre-pregnancy check-up; primary health care ultrasound screening; nationwide newborn screening for congenital hypothyroidism, G6PD deficiency, phenylketonuria and galactosemia), and treatment and rehabilitation of the children who are born with defects (surgical operation for cleft lip with/without palate and congenital heart disease) [3].

Our average prevalence rate was the highest among similar studies in various regions of China, such as Hunan (191.84 per 10,000 PIs) from 2005 to 2014; Inner Mongolia (156.1 per 10,000 PIs) from 2005 to 2008; and overall China (145.43 per 10,000 PIs) in 2009 [18, 25, 29]. Our rate was lower than that reported from the United States (29.2 per 1000 PIs) in 2008, and the reported from the Korea (548.3 per 10,000 births, from 2009 to 2010), but higher than the reported from BDs in southern Vietnam (60.2 per 10,000 live births) [30–32]. Actually Hunan, Inner Mongolia and most of the other similar studies in China use a BD monitoring period between 28 weeks of gestation and 7 days after birth; [25, 29] but in the United State the live birth hospitalizations are used in the 2008 Nationwide Inpatient Sample [30, 31]. Li et al. found that the prevalence rate of BDs rose to 291.4 per 10,000 births from 244.2 per 10,000 births after including less than 28 week BDs in analysis of Guangdong BDs in 2007 [28]. Similarly, a study by Wang et al. showed that in a hospital-based survey from the prenatal 20th gestational week to postnatal 7 days, the prevalence of BDs was 232.7 per 10,000 births, which was significantly lower than that from the population-based survey (232.7 vs. 347.4 per 10,000 births, *P* < 0.001) [27]. In summary, the survey monitoring period and survey sample (hospital-based vs. population-based survey) did impact the prevalence rate and the raw number of BDs epidemiology.

The causes of BDs are complex, including a variety of risk factors such as: advanced maternal age, exposures during the pregnancy, geographical location exposures, and race and ethnicity [2, 8, 33]. There are 56 ethnicities in China. Han is the biggest population comprising about 91.5%, and the second largest ethnicity is Zhuang, comprising around 1.3% [21]. Overall, the prevalence rates of BDs among the ethnic minorities in China are not well known. Guangxi is characterized as one of the five autonomous regions in China for their relatively large populations of ethnic minorities (called the “Guangxi Zhuang Autonomous Region”). In our study, we analyzed the ethnic distribution of total BDs and differences of BD prevalence across major ethnic groups. Overall, we found that ethnic minority groups including Zhuang, Yao, Miao, Dong and all others had a lower prevalence rate in BDs as compared to Han, and the difference in the risk was statistically significant. This finding is consistent with the finding from a population-based survey in Inner Mongolia, China conducted from 2005 to 2008, which showed that ethnic Mongols (147.8, 1/10000) were less likely to have BDs than Han Chinese (155.9, 1/10000) [29]. In several studies from the United States there were observed racial differences in the risk of BDs which were postulated to be related to genetic susceptibilities, cultural or social experiences that could modify exposures, or the combinations of genetic susceptibilities and environmental exposures [30, 34–36]. Does this mean ethnic groups in China have different genetic and partially-genetic causes of BDs or that the BDs could be due to their different life-style and environment exposures? Further epidemiologic study will be required to answer these questions.

Most previous Chinese studies showed that prevalence rates of overall BDs were different between rural and urban environments. For example, in the Inner Mongolia study, in the analysis of BDs in the Xinjiang multi-ethnic region, and in the government BDs Prevention Report in 2012, all concluded that a higher incidence of BDs in rural areas than urban areas [3, 29, 37]. However, in the study conducted in Guangxi from 2001 to 2010 in the epidemiology of BDs based on a BDs surveillance system from 2005 to 2014 in Hunan and the analysis of BDs in Hunan 2009, the prevalence of overall BDs in urban areas was significantly higher than that in rural areas [25, 26, 38]. These differences in results likely relate to socio-economic status, level of education, access to health care, exposure to environmental pollution and the life pressures which may be different between urban and rural areas. These factors may have direct or indirect impact on maternal exposures and could impact the prevalence rate of BDs [18, 29, 38, 39]. Future studies from our center will attempt to correlate more information on residential location of the perinatal infant and any relation to ethnicity as a risk factor for the development of BDs.

The top six BD categories covered most BDs (86.8%) in our study. In a study of BDs in Guangxi from 2001 to 2010, the top six BDs were Schridde syndrome, polydactylism, congenital heart disease (CDH), total cleft lip, ear anomaly and hypospadias. This contrasted to the top six in our study which included musculoskeletal BDs as number one, and then cardiovascular, craniofacial, urogenital, other BDs, and neurologic BDs [26].

Thalassemia gene carriers in Guangxi comprise more than 20% of the population [7], which was the major cause of Schridde syndrome. This is a type of haemoglobin-hemolytic disease and was included in other BDs in our analysis. The most serious Schridde syndromes were almost all electively terminated before gestations of 28 weeks in recent years as a result of improvement in the premarital medical examination and at the level of prenatal diagnosis in Guangxi. Even as our study included gestation less 28 weeks, it still resulted in a lower prevalence of Schridde syndrome (15.27 vs. 29.57, per 10,000 PIs) compared to 2001–2010 in Guangxi [26].

The prevalence of CDH was increasing in our report, which is consistent with outcomes of other current studies from within China; and this would likely be due to the use of fetal ultrasound in antenatal care examinations and the improved surveying system online in China supported by the national finances in Maternal and Child Health Care System [3, 18, 28, 40].

## Conclusions

Our study found ethnic differences in the overall risk of BDs and types of BDs. To our knowledge, this is the first report of ethnic differences in the epidemiology of BDs in a hospital-based surveying system with internal quality controls in Guangxi, China. Our study provided a comprehensive description of ethnic differences in the risk of BDs in the most representative city (e.g., geography, composition of population) in Guangxi.

## Abbreviation

BD (s): Birth defect (s)
Cardio: Cardiovascular
CDH: Congenital heart disease
CI: Confidence interval
Cran: Craniofacial
G6PD: Glucose-6-phosphate dehydrogenase
Gensyn: Genetic Syndrome
HBV: Hepatitis B virus
HIV: Human immunodeficiency virus
Multiple BDs: Birth defects affecting ≥2 organ-systems
Mus: Musculoskeletal
NC: North carolina
PI: Perinatal infants
Ref: Reference
Resp: Respiratory
RPR: Rapid Plasma Reagin
RR: Relative risk
Synd/meta: Syndrome and metabolic disease
Uro: Urogenital
USA: United States of America
